# Referral linkages to support pregnant and postpartum individuals with opioid use disorders in Florida: a social network analysis

**DOI:** 10.1186/s13722-026-00675-1

**Published:** 2026-05-08

**Authors:** Amandeep Kaur Ratta, Rafaella Stein Elger, Wouter Vermeer, Tanner Wright, Asa Oxner, Kimberly Fryer, Jennifer Marshall

**Affiliations:** 1 Chiles Center, College of Public Health, University of South Florida, 13201 Bruce B. Downs Blvd, Tampa, FL 33612 USA; 2 Feinberg School of Medicine, Northwestern University, Chicago, IL USA; 3 College of Medicine, University of South Florida, Tampa, FL USA

**Keywords:** Substance use disorder, Perinatal, Social network, Collaborations, Intersectoral coordination

## Abstract

**Background:**

Opioid Use Disorder (OUD) has emerged as a critical public health issue among pregnant and postpartum individuals. To address this concern, it is of utmost importance that healthcare institutions, community-based programs, and other agencies collaborate to improve the support offered to the concerned population in their OUD recovery journey. A social network analysis was conducted to understand collaborations among multisectoral agencies serving or having potential to serve pregnant and postpartum individuals with OUD.

**Methods:**

Collaborations among agencies (*n* = 79), including three Continuous And Data-drivEN CarE (CADENCE) program clinics, in a large Florida county were mapped. A cross-sectional web-based survey was distributed to these agencies to capture inter-agency collaborations, including services provided (e.g., housing, transportation, etc.), interaction frequency, and perceptions of partner agency confidence, dependence, and value. Open-ended questions identified champion agencies and needs of agencies to better serve the population. Network maps of the agencies were generated to characterize the nature of existing collaborations and identify opportunities for strengthening interagency coordination.

**Results:**

Twenty-six out of 79 enlisted agencies (33%) responded, describing connections with 72 enlisted agencies. The social network analysis of total 376 ties among these agencies demonstrated low density (0.108) but high clustering (0.637), indicating a connected core with tightly knit subgroups and cross-sector bridges especially through social services. CADENCE clinics were inter-linked; CADENCE Co-located pediatric/ psychiatric/Maternal-Fetal Medicine clinic was most connected (38 ties), followed by CADENCE Outpatient Prenatal Care clinic (16 ties), and CADENCE Addiction Medicine (9 ties). Collaboration frequency varied, with 34% of referral ties used only once a year or less, 13% used daily, and 17% never used. Agencies reported having confidence in 82% of collaborations, yet more than half (56%) were considered non-essential (no or little dependency) to achieving patient care goals. Qualitative insights emphasized integrated, trauma-informed care, standardized referral pathways and shared data; destigmatization, and harm reduction; simpler resource navigation and supports for housing and transportation.

**Conclusion:**

Overall, the county possesses a multisector perinatal OUD network that is yet underutilized. Strengthening structured referrals, interoperability, and wraparound, stigma-free services leveraging CADENCE could reduce fragmentation and improve maternal-infant outcomes.

**Trial registration:**

ClinicalTrails.gov (NCT05609669). November 02, 2022.

**Supplementary Information:**

The online version contains supplementary material available at 10.1186/s13722-026-00675-1.

## Background

The opioid crisis has reshaped the landscape of maternal and child health in the United States, with Opioid Use Disorder (OUD) emerging as a critical public health concern among pregnant and postpartum individuals [1, 2]. Among peripartum populations, OUD is closely intertwined with structural and socioeconomic factors, including economic instability, residence in under-resourced communities, housing insecurity, and fragmented access to care, including delayed and inadequate prenatal care [3–5]. From 1999 to 2014, the prevalence of OUD at the time of delivery hospitalizations increased significantly, rising from 1.5 cases per 1,000 deliveries in 1999 to 6.5 per 1,000 deliveries in 2014 [2]. This increase was even more pronounced in Florida, escalating from 0.5 per 1,000 in 1999 to 6.6 per 1,000 in 2014 [2]. Although the prevalence of OUD during pregnancy in Florida is comparable to the national rate, the state had a higher drug-related mortality ratio among non-pregnancy-related deaths (NPRD) from 2009 to 2019 (20 vs. 7.8 per 100,000 live births) [6, 7]. Furthermore, studies from Florida have identified multiple barriers to care among pregnant individuals with OUD, including financial constraints, insurance-related limitations, and challenges in accessing timely prenatal care [8, 9]. In addition, evidence points to missed opportunities for accurate identification and diagnosis of OUD in maternity hospitals at the time of delivery [10], underscoring the need for improved care coordination and quality-focused interventions for this population.

This study was conducted in Hillsborough County, Florida, United States, a predominantly urban county within a metropolitan area. The county has a population exceeding 1.5 million residents and is racially and ethnically diverse, with approximately 44% identifying as non-Hispanic white, 32% Hispanic or Latino, and 18% Black or African American [11]. Despite the presence of multiple health care systems and safety-net providers [12], access to behavioral health and substance use treatment services remains uneven, particularly for pregnant individuals requiring medication for opioid use disorder (MOUD) and coordinated prenatal care [8, 9, 13].

OUD during pregnancy can significantly increase risks for both the mother and infant. Potential consequences include neonatal opioid withdrawal syndrome (NOWS), preterm birth, stillbirth, low birth weight, neonatal intensive care admission, involvement with child protective services, and maternal overdose [14, 15]. Medication for opioid use disorder (MOUD) is linked to better prenatal care adherence and improved birth outcomes [16]. However, women encounter several barriers to accessing these services, including transportation, time off work, stigma, and challenges in navigating the healthcare system (e.g., obtaining appointments for prenatal care [9] and opioid use treatment [13]), requirements for daily dosing or inpatient treatment [17], co-occurring mental health conditions, limited family support, and unstable housing [18]. A study in Florida found that pregnant women with OUD faced significant barriers to establishing care with substance use and mental healthcare providers [8]. In addition to MOUD, establishing timely prenatal care and attending visits consistently, including during the postpartum period, are associated with improved maternal and neonatal health outcomes [17]. Studies suggest that individuals with perinatal OUD initiate prenatal care later and experience disruptions in both prenatal and postpartum care engagement, contributing to poor maternal and infant outcomes and highlighting the need for coordinated, integrated care models [3]. Another study found that overdose-related deaths increased during the COVID-19 pandemic in this population, highlighting the need for stable, integrated healthcare systems [19]. An ideal model of care for this population should integrate OUD treatment (including MOUD, counseling, and psychiatric services) with standard prenatal visits [17].

In addition to clinical care, pregnant patients with OUD should receive holistic services to address trauma, mental health, and social and economic needs, which can improve outcomes such as reduced return to use and positive perinatal outcomes [20–22]. It is crucial that pregnant patients with OUD receive integrated care from a multidisciplinary team of physicians, social workers, therapists, and case managers [22], effectively addressing patients’ social needs and facilitating access to community resources [23, 24]. Such measures may also reduce costs related to foster care for children of mothers with OUD and NOWS-related readmissions [20]. Integrated services help prevent return to use by addressing pregnancy-related complications, perinatal depression, abuse, poverty, stigma, and legal concerns, which may be exacerbated by inadequate support systems and poor coordination of care [25]. Access to programs that offer or refer to these services is limited, as shown in the National Institute on Drug Abuse Medication Treatment for Opioid-dependent Expecting Mothers study, which revealed that while over 75% of participating sites provided medical and behavioral health care, less than half offered services such as childcare and housing [26]. There are also challenges in implementing programs that follow this integrated approach due to a lack of external support and resources, as patients may be unable to attend appointments or access residential treatment programs because of transportation barriers and geographic distance [23]. Moreover, the absence of standardized mechanisms for coordination between residential and outpatient providers offering MOUD has been identified as an additional barrier to supporting pregnant patients [23].

To address gaps in coordinated care for pregnant individuals with OUD, the Continuous And Data-drivEN CarE (CADENCE) program was developed as part of an NIH-funded initiative (2022–2027) to improve maternal and infant outcomes through integrated service delivery [27]. The program was conceptualized by a multidisciplinary team of clinicians at the three CADENCE intervention sites, researchers, and public health professionals at a state-level academic institution in Florida to address fragmented access to prenatal care, MOUD, pediatric care, and behavioral health services. CADENCE integrates clinical care coordination with a data-driven approach to monitor maternal, neonatal, and infant outcomes and support continuous quality improvement. Key components also include the development of an interactive dashboard to track outcomes among pregnancies affected by OUD and to evaluate the implementation and cost of the integrated care model. By coordinating care across clinical and community settings, this program aims to improve maternal engagement in treatment, reduce neonatal opioid withdrawal syndrome, and strengthen long-term outcomes for mother-infant dyads affected by OUD.

Currently, this program is being implemented at three sites in Hillsborough County: the CADENCE Addiction Medicine Clinic, the CADENCE Outpatient Prenatal Care Clinic, and the CADENCE Co-located Pediatric/Psychiatric/Maternal-Fetal Medicine Clinic. These sites were existing clinics that were subsequently integrated under the CADENCE model to enhance coordination across services. No specific clinic names are presented here to preserve confidentiality.

Strengthening collaboration between healthcare institutions, community-based programs, and social service organizations is essential for supporting pregnant and postpartum individuals throughout their OUD recovery journey. As an integrated care initiative, CADENCE relies on effective coordination among healthcare providers and community agencies to connect patients with clinical services and social supports. Understanding how these organizations collaborate and refer patients is therefore critical for identifying opportunities to improve care coordination within the program. To examine these relationships, a social network analysis was conducted to assess collaborations and referral patterns among key agencies providing services to this population. This was done by (1) identifying connections among agencies providing healthcare and social services to pregnant and postpartum individuals with OUD, including the CADENCE clinics, and (2) identifying key agencies that serve as champions for coordinated, holistic care for this population.

## Methods

This cross-sectional study used social network analysis (SNA) to understand collaborations and referrals among key agencies involved in providing or having the potential to provide healthcare and social services (e.g., food, housing, transportation, legal services, etc.) to pregnant and postpartum individuals with OUD in Hillsborough County, Florida.

SNA is a quantitative methodology used to analyze structural properties and interactions among a wide range of agents. By representing individuals or organizations as vertices and their relationships as edges within a graph, such a representation can be used to explore how information, resources, and referrals flow across a service system [28, 29]. Key network metrics include degree distributions (number of direct connections an agency has), density (proportion of all possible ties that are actually present), and clustering coefficient (the degree to which an agency’s partners are connected to each other), which reflect influence, cohesion, and subgroup connectivity within the network [28, 30, 31]. Network findings are reported following conventions used in published interorganizational SNA studies, including network level-metrics, node-level centrality, and network maps [30–32].

SNA has been increasingly applied in health and social services research to examine interorganizational collaboration, identify gaps in care coordination, and detect fragmentation in service delivery [32, 33]. Within substance use and related behavioral health research, SNA has been used to examine service coordination among addiction treatment providers, map referral networks for individuals with co-occurring substance use and mental health disorders and assess the structural capacity of community coalitions addressing substance use [34–36]. This approach is particularly appropriate for the present study, as it enables identification of highly connected agencies and key actors, assessment of overall network cohesion, and detection of gaps or fragmentation in referral pathways, all of which are critical for understanding coordinated care access for populations with complex needs such as pregnant and postpartum individuals with OUD.

The survey instrument used to capture network data was developed using a mix of validated SNA constructs drawn from established interorganizational network research [37, 38] and items developed by the study team to reflect the specific context of perinatal OUD care. Survey items captured multiple dimensions of interorganizational relationships: presence of referral linkages, frequency of interaction, and perceived partner confidence, trust, and value, constructs commonly used in SNA of health and substance use service networks to assess both structural and functional aspects of collaboration [37, 38]. The questionnaire was pre-tested among study team members. The study was reviewed and determined exempt by the [blinded for peer-review] Institutional Review Board.

A total of 79 agencies providing or having potential to provide healthcare and social services to this population were identified, and each was considered as a measurement unit. These agencies were identified through a multi-step project that included (1) consultation with CADENCE project leadership and clinical partners, (2) Review of existing resource guide and community partner directories that were already in place as a part of the CADENCE project, and (3) iterative refinement through team discussion to ensure inclusion of agencies providing or having potential to provide services to pregnant and postpartum individuals with OUDs. These agencies were categorized into sectors based on their primary service domain using predefined criteria informed by prior literature on integrated care and community-based service delivery, for example, an agency providing housing support would fall under the social services type [39]. The categories were as follows: substance use and mental health, social services, support groups, judicial/child welfare, healthcare (obstetrics and gynecology), healthcare (pediatrics), and healthcare (multiple, other). Three CADENCE clinics were included within these 79 agencies as distinct entities and represented key intervention sites within the network. These were categorized as follows: CADENCE Addiction Medicine Clinic, CADENCE Outpatient Prenatal Care Clinic, and CADENCE Co-located Pediatric/ Psychiatric/Maternal-Fetal Medicine clinic. No specific clinic names are presented here to preserve confidentiality. These clinics were included in the survey and contributed data in the same manner as all other participating agencies.

### Data collection and analysis

Data were collected via an internet-based survey distributed through Qualtrics [40] and contained closed-ended questions related to network connections and open-ended questions to gather information on the agencies’ views and perceptions of collaboration (Supplementary file – Appendix I). The survey began with an overview of the study’s purpose and a request for consent to participate. Respondents were also given the option to mention any additional agencies they collaborated with apart from the 79 listed agencies.

A flyer with the survey link was emailed to all identified agencies. Up to six reminder emails were sent at approximately 2-week intervals to improve response rates. In cases where initial contacts were unresponsive, efforts were made to identify alternate contact information to reattempt outreach. Principal Investigators and the CADENCE Clinic leads helped with reaching out to the agency level contacts and encouraging them to fill out the survey. Each agency was asked to provide a single response representing their organization. Responses were accepted from any knowledgeable staff member familiar with referral practices and collaborations within their agency. Responses were collected from January to June 2024. Of these 79 identified agencies, 13 could not be reached due to invalid or outdated contact information or lack of publicly available contact details. Efforts were made to reach out to these agencies by filling out the online forms available on their websites to reach out to them, and/ or by calling the phone numbers available publicly. These agencies were retained in the sampling frame but excluded from analyses requiring survey responses. Their inability to be contacted may reflect structural gaps in interagency communication within the network. While identifying the agencies from the given list of 79 agencies, participants were given the option to add any additional agencies they networked with. These agencies not included in the initial list were documented for descriptive purposes. However, they were not included in the primary network visualizations, as they were not a part of the original survey administered to all agencies, and therefore lacked completed network-level data. Including those agencies in the network visualizations would affect network integrity. Connections involving these agencies were retained for descriptive analyses where appropriate.

All 79 agencies were retained as nodes in the network. Non-responding agencies were included as nodes if mentioned as collaboration partners by at least one responding agency, allowing their structural position to be characterized based on incoming ties. Agencies neither responding nor mentioned by respondents appear as isolates in the network maps. Network data were structured based on reported collaboration ties. Relationships were treated as undirected, such that a connection was considered present if either agency reported a formal or non-formal relationship in the survey. Network metrics included degree centrality, density, and clustering coefficient and were computed using R and visualized using Gephi. Each agency is represented by a numbered node, color-coded according to the sector it belongs to (detailed in Figs. 2 and 3). Agencies marked by squares instead of circles highlight the three CADENCE clinics, emphasizing their distinct role within the network. The lines connecting different nodes represent a link or relationship, defined in the social network map as “any type of relationship indicated”. The size of the nodes indicates their degree, the number of connections an agency has; larger nodes denote agencies with more connections and can be considered key players within the network.

The data were exported into Microsoft Excel (Version 2406 Build 16.0.17726.20078). Two researchers examined the data to check for any errors and inconsistencies, and the clean dataset was imported into R software (version 4.3.3) for data manipulation [41] and subsequently exported to Gephi (version 0.10.1) for further analysis [42]. Microsoft Excel was used to analyze the demographic characteristics of participating agencies and compile the responses for the two open-ended questions, which were then summarized. Response to the open-ended question on community needs to improve holistic care for individuals with OUD were reviewed and summarized narratively using a qualitative descriptive approach. One researcher reviewed all responses and grouped them into recurring topics, which were then organized into broader themes reflecting common patterns in the data. A senior researcher reviewed the thematic summaries to ensure consistency and credibility of interpretations. Discrepancies were resolved through discussion and consensus.

This study was registered with ClinicalTrials.gov (NCT05609669) on November 02, 2022.

## Results

**Fig. 1:**
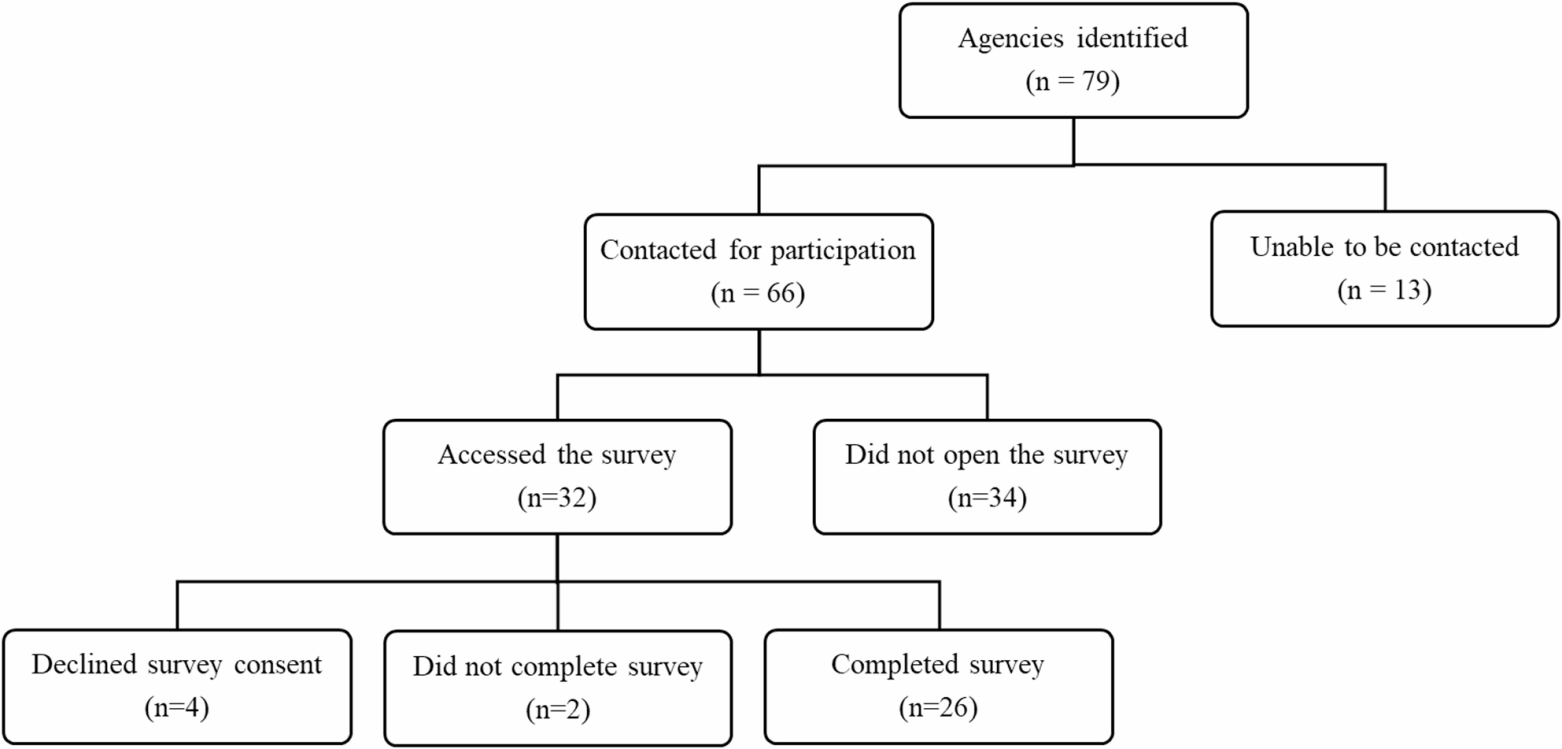
Flowchart of agency participation in the survey

In total, 79 agencies that potentially served pregnant and postpartum individuals with OUD in the county were identified. Of those, 26 including three CADENCE intervention sites completed the survey, resulting in a response rate of 33% (Fig. 1). The survey questionnaire included all 79 identified agencies, allowing participants to identify connections with each. Survey respondents included program directors, coordinators, managers, physicians, and administrators, and 50% of the agencies were part of larger organizations. Participating agencies represented a range of sectors, with the majority from social services (*n* = 11), substance use and mental health (*n* = 5), and healthcare (obstetrics and gynecology) (*n* = 3) (Table 1).

Participating agencies that completed the survey identified referral linkages and collaboration with 72 of the 79 listed agencies. Hence, final analysis included the identification of referral linkages and collaborations identified by 26 participating agencies among 72 agencies in the county. Additionally, the survey allowed agencies to list agencies not included in the original list for future surveys; five additional agencies and local providers with whom they collaborated were noted. However, those additional agencies were not included in the network maps as they were not part of the original network survey sent to all participants.

**Table 1:** Overview of identified, participating, and non-participating agencies by sectors

Sector	Agencies identified (*N* = 79)	Completed survey (*N* = 26)	Did not complete the survey (*N* = 53)
Substance use and mental health	25	5	18
Social services	27	11	16
Support groups	5	1	4
Judicial/child welfare	3	1	2
Healthcare – obstetrics and gynecology	6	3	3
Healthcare – pediatrics	8	2	6
Healthcare – multiple disciplines	5	3	2

The total number of patients or clients served annually by the participating agencies ranged from zero to 120,000. The one agency reporting zero patients functioned in a funding and coordination role and did not directly provide services. Excluding this agency, the proportion of pregnant or postpartum patients among all patients served annually ranged from 0% to 100% (mean 26.8%). The presence of 0% value reflects agencies whose primary focus (e.g., pediatric care, substance use disorder treatment, or peer support) does not involve pregnant populations. Similarly, the percentage of pregnant or postpartum individuals with OUDs served ranged from 0% to 100% (mean 28.2%), with some agencies serving pregnant populations, but not specifically pregnant individuals with OUDs. The agency with no direct patient population was still included in the network analysis because other agencies identified ties with it and removing it would affect the network integrity.

As part of the survey, respondents were given a list of services and asked to identify which of them they provided to pregnant and postpartum individuals with OUD (Table 2). Referral to agencies providing OUD services was reported by 18 (64%) agencies. Other commonly reported services included OUD counseling/recovery (50%), OUD management/recovery (42%), and postpartum care (46.4%). Less commonly reported services included housing (3.6%), ambulance services (3.6%), and legal services (0%).

**Table 2:** Services provided by survey respondents

Type of service provided	Number of responding agencies who provide this service	
	*n*	%
Referral to agencies providing OUD services	18	69
OUD counseling/recovery	14	54
Postpartum care	13	50
OUD management/recovery	12	46
Pregnancy care	10	38
Child development	9	35
Pediatric care	7	27
Peer-to-peer support groups	7	27
Others ^a^	7	27
Care during childbirth	4	15
Transportation	4	15
Nutrition/food resources	4	15
Family violence	3	11
Needle exchange	2	8
Housing	1	4
Ambulance service	1	4
Legal services	0	0

*Notes*: *N* = 26^a^ Other services mentioned by the participating agencies were linkages to community services, nurturing parenting program, direct peer recovery services, information and referral services, HIV testing and medical care

In an open-ended question, participants were also asked to identify the top three champion agencies in their community to whom they went for information on evidence-based practices for OUD. The most frequently mentioned included an agency focused on substance use and mental health services mentioned by 10 respondents, followed by the CADENCE Co-located Pediatric/Psychiatric/Maternal-Fetal Medicine Clinic (*n* = 8), and an agency providing social services support, and education (*n* = 4). Through second open ended question, agencies identified key areas for improving holistic care for people with OUD: (1) access to care, (2) coordination of care and collaborations among agencies, (3) need for community education on destigmatization of the OUDs, (4) increased availability of mental health resources, (5) provider training in trauma-informed care, (6) support for social needs such as housing, transportation, and (7) development of a centralized compendium of local resources.

### Social networks

**Fig. 2:**
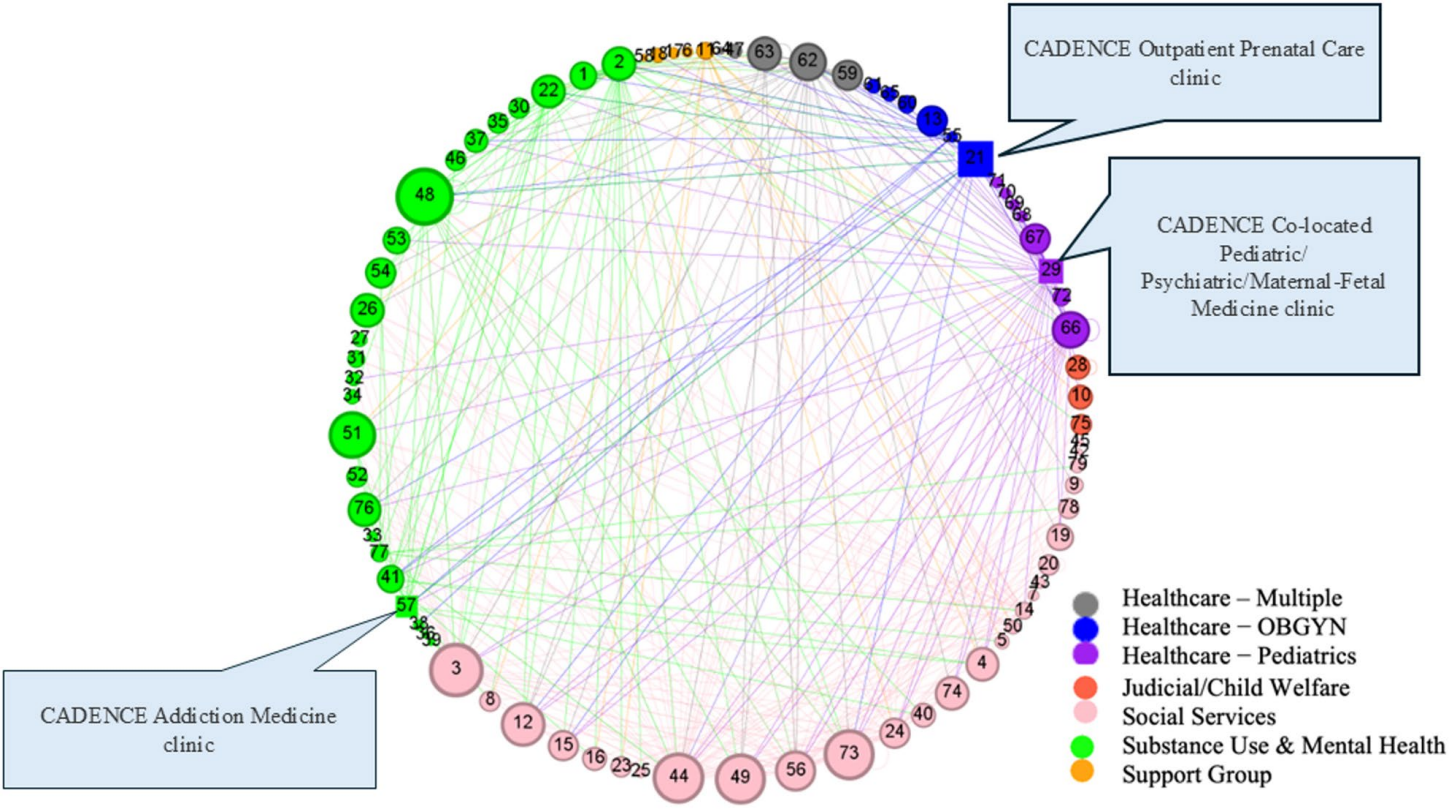
Network map showing all agency linkages for referral services

**Fig. 3:**
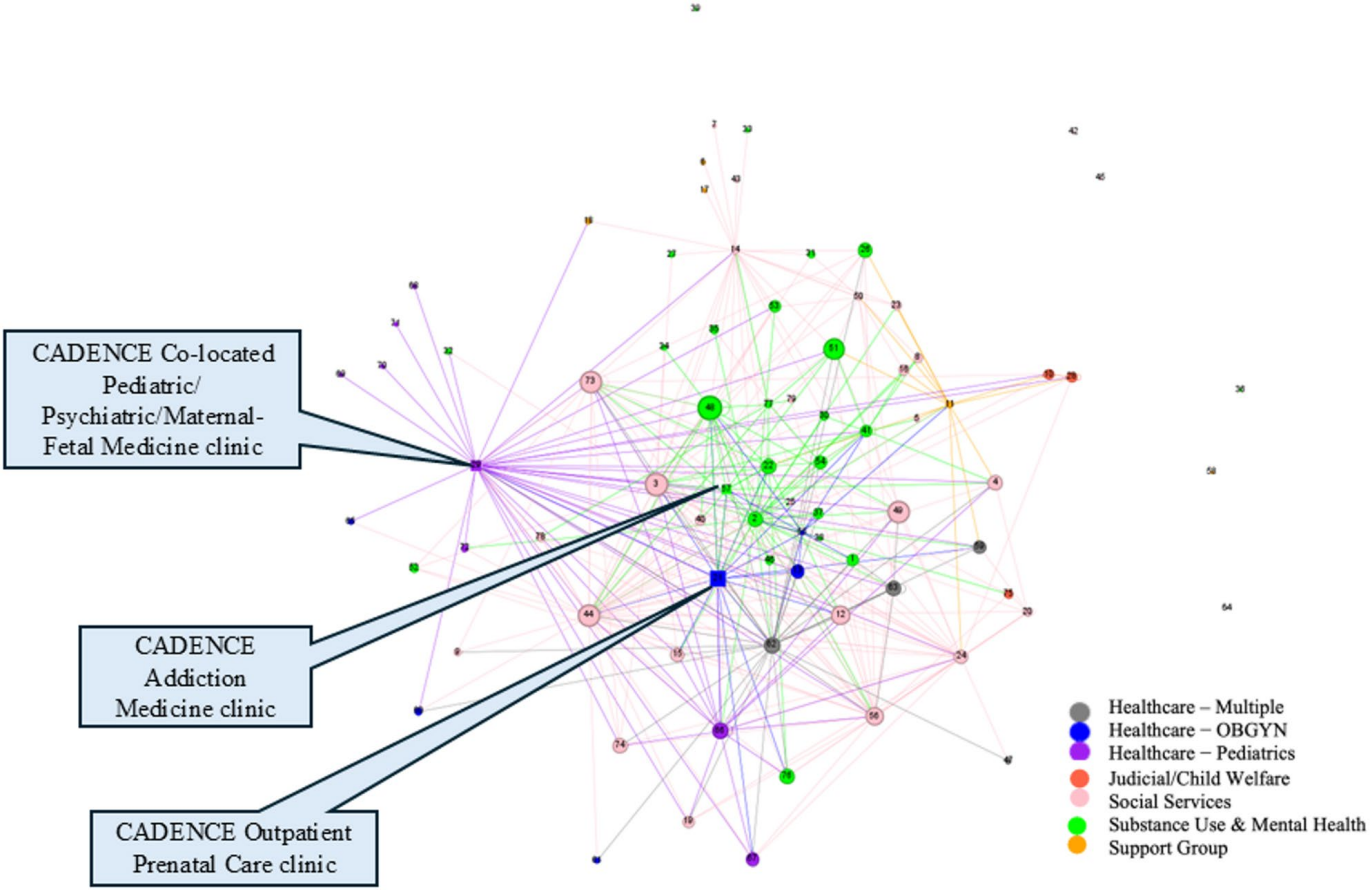
Network map – All ties

Network maps were developed based on survey responses to illustrate collaboration and referral relationships among the 79 initially identified agencies. Figures 2 and 3 illustrate the collaboration patterns among agencies providing services to pregnant and postpartum individuals with OUD.

Each agency is represented by a numbered node, color-coded according to the sector it belongs to, with node size proportional to degree centrality (detailed in Figs. 2 and 3). A total of 376 ties were identified among the 73 agencies as reported by 26 respondents in the network. Six agencies neither participated, nor were identified as a collaborator by any of the respondents are represented as standalone nodes in the maps.

### Overall connectedness

The network demonstrated a relatively low density (0.108) but relatively high average clustering coefficient (0.637), suggesting strong collaboration among groups of connected organizations, with ample opportunity to further increase connectivity and improve collaboration and integration. We found no indication of further clustering within the network map, indicating that the system as a whole is relatively well-connected. Additionally, no insulated subgroups are present, resulting in a well-connected core, which is surrounded by more peripheral organizations. When organizing by sector (Figs. 2 and 3), we observed a significant number of cross-sector connections, particularly within the social services sector, further supporting the observation that this network is relatively well-connected.

CADENCE Co-located Pediatric/ Psychiatric/Maternal-Fetal Medicine clinic **(node 29)**, was identified as one of the key players in the network, receiving and sending a high number of referrals from other healthcare providers, social service agencies as well as support groups. High connectivity of the Healthy Start Coalition of the county **(node 44)** reflected the importance of social services in supporting pregnant and postpartum individuals with substance use disorders and their children.

### CADENCE intervention sites’ connectedness

All three CADENCE intervention clinics indicated connections with each other. CADENCE Co-located Pediatric/ Psychiatric/Maternal-Fetal Medicine clinic **(node 29)**, serving neonatal and pediatric populations, was found to be most connected, referring to and receiving patients from all types of agencies (*n* = 38), followed by the CADENCE Outpatient Prenatal Care clinic **(node 21)** having connection with many of the agencies (*n* = 16) across groups (except support groups). The CADENCE Addiction Medicine clinic **(node 57)** noted connections with nine agencies providing OBGYN, substance use & mental health, and social services.

### Engagement and trust within networks

**Fig. 4:**
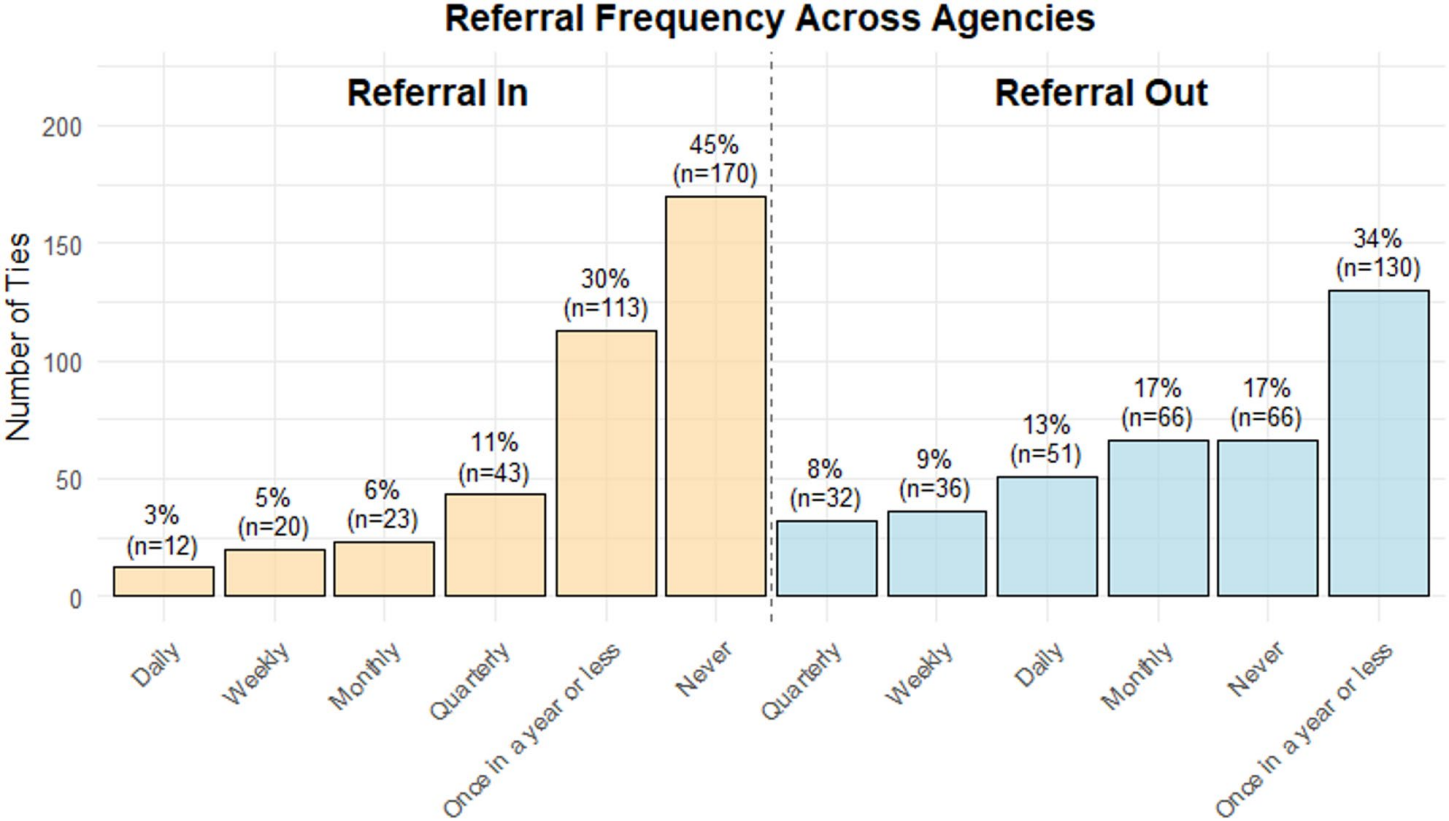
Referral frequency across agencies

The survey also focused on engagement frequency and trust within the network. Participants were asked about the frequency of sending and receiving referrals for patients from the agencies with which they had relationships. While network maps illustrate 376 collaborations among 26 participating agencies, participants mentioned five additional agencies. Although not included in the network visualizations, these five agencies were included in frequency and trust analyses. Therefore, the findings below are based on 381 (376 + 5) ties. Of the 381 identified connections, 26 agencies referred patients to 34.1% of connections only once a year or less, while they referred outpatients to 13.4% of the connections daily. They never referred patients to 17.3% of the identified connections (Fig. 4). On the other hand, they mentioned that they never received referrals from 44.6% of the identified connections, received a referral once a year or less from 29.7% of the connections, and received daily referrals from 3.1% of the connections. (Fig. 4).

Although formal stratified analyses by agency type were not conducted, visual inspection of the network maps (Figs. 2 and 3) suggests that healthcare and social services agencies tended to occupy more central positions within the network. In contrast, some specialized organizations appeared to have fewer connections or no connections (some standalone nodes in Fig. 3), reflecting variations in roles and levels of integration within the service system.

**Fig. 5:**
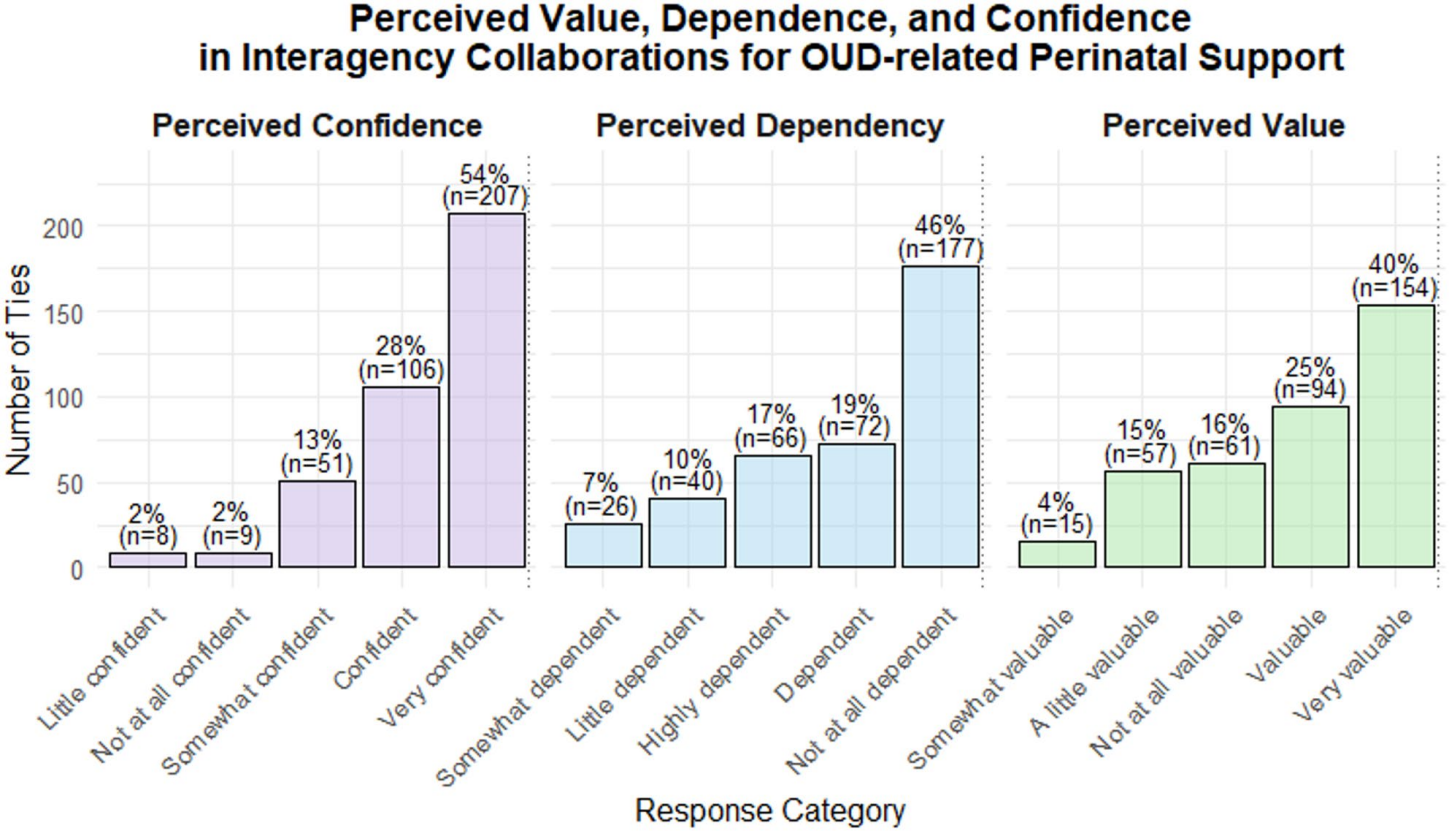
Perceptions about interagency service provision to pregnant and postpartum individuals

Participants were asked how confident their agency was in collaborating agencies’ ability to provide evidence-based services to the clients referred by them. Agencies reported high confidence (combining “very confident” and “confident”) in the majority of collaborations (82%), while a low confidence (combining “a little confident” and “not at all confident”) was reported in small proportion (4%) (Fig. 5).

When asked how dependent their agency was on each collaborating agency for achieving their goal of helping pregnant and postpartum individuals with OUDs, agencies perceived themselves as being dependent (combining “highly dependent” and “dependent”) in 36% of collaborations. However, no or a little dependency was reported for more than half (56%) of collaborations (Fig. 5). Participants also rated how valuable each agency’s involvement was for their own agency’s success in supporting pregnant individuals with OUDs. Collaborating agencies were considered valuable (combining “very valuable” and “valuable”) in more than two-thirds (65%) of collaborations, while 31% of collaborations were viewed as a very little or not at all valuable (Fig. 5).

### Qualitative findings

Participants were also asked to share their perspectives on what was most needed in the community to improve holistic care for people with opioid use disorders and prevent overdose. Out of the 26 responding agencies, 22 (85%) provided responses to this question. They emphasized the need for integrated, trauma-informed, and coordinated care approaches that address both medical and social determinants of health. Key areas identified included expanding mental health services, reducing stigma, meeting basic needs (housing and transportation), and ensuring affordable treatment options. Importance of raising awareness and improving information about available resources was also highlighted. These findings underscore the importance of a holistic, person-centered approach to OUD recovery. Common themes along with summaries, and representative quotes are presented in Table 3.

**Table 3:** Quotes related to agency identified needs to enhance holistic care for opioid use disorders in the community

Theme	Summary of findings	Quote
Integrated and coordinated care	Need for integrated service delivery, such as co-located physical and behavioral health services, to provide comprehensive care within one setting.	“I would say the move to a community behavioral health clinic where the patients can receive both primary care and behavioral health care in one place.” - Substance use and mental healthcare provider
Resource awareness and accessibility	Need for centralized, accessible information and improved system navigation with consistent messaging across agencies.	“A compendium of local programs/resources available to community members.” - Substance use and mental health provider “More universal and consistent messaging - from a central source that focuses on messaging, connecting, and being a liaison for the systems of care.” - Social services provider
Community education and destigmatization	Need for community education, including among healthcare providers to reduce stigma, normalize recovery, expand access to harm-reduction resources such as naloxone, and promote trauma-informed training for providers serving postpartum individuals	“Education in the community reducing stigma. Education and advocacy messaging that recovery looks different for everyone. Education regarding the poisoning with fentanyl in the drug supply. Narcan everywhere and in all First Aid Supply boxes.” - Support group “More providers who are trained in trauma informed care who can work with the postpartum-newborn dyad.” - Pediatrics healthcare services provider
Access to housing and transportation	Stable housing and reliable transportation identified as prerequisites for engaging in treatment or maintaining recovery	“Housing and reliable transportation, more resources for those unhoused and with unreliable transportation. It is difficult to treatment someone for addiction/expect someone to discontinue the use of opioids and other substances when they are unstable in their life. their hierarchy of needs should be met and we struggle with lack of resources/availability of the current resources.” - Substance use and mental healthcare provider
Affordable and accessible treatment options	Need for expanded, affordable treatment options, including mobile clinics and free or low-cost treatment services	“Mobile Suboxone clinics for free. Free chiropractic care. Free treatment through private treatment centers. Psychological treatment availability. Trauma informed care.” - Social services provider
Mental health resources	Need for expanded mental health services, particularly inpatient treatment options for co-occurring mental health conditions that often accompany the OUDs	“More options for inpatient treatment for mental health conditions precipitating the opioid addiction.” - Multiple healthcare services provider

## Discussion

This study examined the collaborations among a network of agencies providing healthcare and social support services to pregnant and postpartum individuals with OUDs in a large Florida county. Findings revealed substantial cross-sector connections. However, low network density indicated opportunities to strengthen interagency collaborations to better support this population, while limited engagement among many agencies suggests a need for improved coordination.

### Network strengths and areas for improvement

Given that CADENCE was designed as an integrated care model to improve coordination across clinical and community services, the positioning of its clinics within the broader referral network provides important context for interpreting the strengths and gaps observed in the interagency collaboration.

The social network mapping revealed a well-connected core within the network, characterized by strong cross-sector collaboration. Social services agencies, in particular, demonstrated high levels of intra-sector collaboration, aligning with previous studies highlighting their critical role in supporting pregnant individuals with substance use disorders [43]. The network’s high average clustering coefficient (0.637) further reflected the presence of tightly knit collaborations among a small group of organizations within the broader network structure [44]. Focusing on the three CADENCE clinics (described in detail in the background section), the survey highlighted varying levels of connectedness within the network. CADENCE Co-located Pediatric/ Psychiatric/Maternal-Fetal Medicine clinic, emerged as the most connected site, maintaining referral relationships with agencies across all service sectors (*n* = 38), followed by CADENCE Outpatient Prenatal Care clinic, demonstrating ties to 16 agencies spanning OBGYN, substance use and mental health services, and social services agencies, although no reported linkages with peer support groups were noted at this early stage. The CADENCE Addiction Medicine clinic, primarily focused on substance use disorder treatment, exhibited referral connections with nine agencies, primarily those offering OBGYN, mental health, and social services. While all three CADENCE clinics reported connections with one another, the degree of collaboration at the time of the survey reflects the early phase of CADENCE implementation. The emphasis during this period was on establishing a data dashboard system to support care coordination. As the CADENCE program progresses into its clinical process integration phase (years 4 and 5), it is anticipated that inter-clinical referral pathways and cross-sector collaborations will strengthen, promoting a more cohesive network across CADENCE sites and community partners. Participants also identified trusted champions across diverse sectors, including substance use and mental health services, pediatric care, and social services, highlighting the importance of multisectoral collaboration in promoting best practices for OUD care for pregnant and postpartum individuals [26].

Despite the strength of these core relationships, the overall network density was low (0.108). In network terms, it reflects that out of all possible ties among the 79 enlisted agencies, only 10.8% were active at the time of survey as reported by the participating agencies [30, 31]. Although more connections might exist among non-participating agencies, based on the reported ties, it suggests limited connectivity across the network as a whole, indicating opportunities to strengthen relationships between agencies. Furthermore, some participating agencies reported low confidence in certain partnerships, which may reflect concerns regarding service quality, the absence of standardized referral processes, or insufficient communication between organizations [26]. Additionally, the frequency of collaboration varied significantly. While some agencies engaged in daily partnerships, others reported minimal or no interactions at all. This could be due to the overall population served by the responding agency, which may include a relatively small number of pregnant or postpartum patients, or patients with OUDs. Such inconsistencies may contribute to fragmented care experiences for individuals with OUDs, demonstrating the need for more coordinated and consistent collaboration across systems of care [23] and suggests a need for increased outreach to improve awareness and training for serving this population.

In addition to mapping inter-agency connections, this study explored how participating agencies perceived the quality of their collaborations. While agencies expressed high confidence in majority of their collaborations and rated many connections as valuable, nearly half reported no dependence at all on these collaborations. Similarly, agencies referred patients out only once a year or never in 51.4% of their collaborations and never received a patient from 44.6% of the identified connections. These findings align with previous research indicating that even well-connected networks do not necessarily foster frequent collaborations, trust, or inter-agency service utilization [37, 45]. Although many agencies valued their partnerships and expressed high confidence in service delivery which might reflect relational trust [46, 47], the high proportion of ties with limited or no referrals suggests underutilized collaborations or siloed service delivery, consistent with patterns observed in other behavioral health networks [48, 49]. These findings highlight the importance of referrals in fostering successful collaborations to improve care coordination and outcomes for pregnant and postpartum individuals with substance use disorders, as emphasized in previous research [50].

Additionally, survey respondents emphasized the need for holistic, trauma-informed, stigma-free, and integrated care models to support people with substance use disorders during the perinatal period. They also emphasized the importance of access to social services such as housing, transportation, etc., for successful recovery from substance use disorders. The need for addressing social needs among pregnant individuals with substance use disorders has also been expressed in other scholarly work [51]. Consistent with prior literature on OUD treatment, participants reported challenges related to awareness of resources and the complexity of navigating the healthcare systems [52]. They advocated for centralized resource guides and unified messaging to support patients seeking treatment and recovery services. Stigma, as identified in other studies [53], emerged as a major theme, with participants calling for community education and provider training in trauma-informed care. Overall, these insights underscore the importance of coordinated care, community engagement, and structural support systems in promoting equitable and sustained recovery for pregnant and postpartum individuals affected by OUD.

### Strengths and limitations

To our knowledge, this is among the first studies in Florida to examine multisectoral collaborations for supporting pregnant and postpartum individuals with OUD. Prior studies from other U.S. settings have described integrated care models and collaborative approaches for this population, including community-based programs and multisite clinical collaborations [20, 54, 55], however, few studies have examined interorganizational relationships across healthcare and social sectors using network-based methods for this particular population in the country. We identified a large number (79) of potential agencies in one county. This provides an important contribution to the growing literature highlighting the importance of collaborations among healthcare and non-healthcare sectors in addressing complex health and social needs [56]. Also, this study captured not only the presence of referral ties but also their perceived value, and included open-ended questions related to community champions and unmet community needs. The survey also functioned as a tool to evaluate CADENCE clinics’ inter-sectoral and inter-agency integration.

One limitation was the low response rate. The results were also limited in that participants were asked to rate the value of their inter-agency connections but not prompted with follow-up questions to further describe the reasons behind their responses. Survey respondents may not have had knowledge of how many clients with perinatal OUD their program or organization serves, the types of services provided, all referral relationships, or the frequency of those referrals. There may have been social desirability biases in survey responses. Furthermore, analyses of trust/ confidence ratings by agency type were not conducted due to the small number of responding agencies within each sector, which precluded meaningful subgroup comparisons. There may be additional programs or key partners we are unaware of, hence those may not have been captured in this study. Future research could build on these findings by incorporating qualitative approaches to better understand the factors influencing perceived value and trust in collaboration.

### Implications for practice

This study underscores the importance of moving beyond network connectivity toward fostering more meaningful, frequent, and coordinated collaboration among agencies serving pregnant and postpartum individuals with OUDs. While many agencies valued their partnerships, the infrequent referrals and limited interdependence observed suggest opportunities to strengthen systems of care. Efforts to improve collaboration should prioritize establishing clear referral pathways, improving communication between agencies, and addressing structural barriers that limit service coordination. Several strategies may strengthen interagency connectivity and collaboration quality in networks like the one explored in this study. Literature suggests that network development can be supported through formal referral protocols, shared data platforms, regular cross-agency shared decision-making meetings, regular interagency trainings, shared resource directories, bridging/ hub organizations, and governance structures that promote trust, accountability, and information exchange across sectors [55, 57, 58]. Programs like CADENCE illustrate the potential of integrated, multisectoral approaches that combine clinical care with social supports to better meet the complex needs of this population. Future practice should focus on fostering trust-based relationships across sectors, investing in infrastructure that facilitates collaboration, and using local data to guide resource allocation and service delivery. Ultimately, improving care for pregnant and postpartum individuals with OUDs will require intentional, sustained collaboration across healthcare and social service systems to reduce fragmentation and ensure more equitable access to comprehensive care.

Future research should examine how this network evolves as CADENCE moves further into clinical integration and whether increases in interagency connectivity are associated with more timely referrals, greater service uptake, and improved maternal and infant outcomes [58]. Longitudinal network analyses, follow-up interviews with key agencies, and stratified analyses by agency sector [44] could help clarify which relationships are most influential for coordinated perinatal OUD care. Additional work is also needed to evaluate whether interventions such as shared referral pathways, patient navigation, and strong community resource infrastructures improve both network performance and patient outcomes [29, 55].

## Conclusions

In summary, this study provides valuable insights into the collaborative landscape among agencies supporting pregnant and postpartum individuals with OUDs in a region in Florida with a high number of births and prevalence of opioid exposures during pregnancy [59]. Strengthening multisectoral partnerships, particularly between healthcare and social service agencies, is essential to improving care coordination and outcomes for this population. This study contributes to the existing literature emphasizing the importance of cross-sector collaboration to address the complex needs of individuals with substance use disorders [56].

## Supplementary Information

Below is the link to the electronic supplementary material.

Supplementary Material 1

## Data Availability

De-identified datasets used and/or analyzed during the current study are available from the corresponding author on reasonable request.

## Abbreviations

CADENCE: Continuous and Data-Driven Care
MOUD: Medication for opioid use disorder
NAS: Neonatal Abstinence Syndrome
NOWS: Neonatal Opioid Withdrawal Syndrome
OUD: Opioid Use Disorder
