# Supplementary material for: Referral linkages to support pregnant and postpartum individuals with opioid use disorders in Florida: a social network analysis
Source: Addict Sci Clin Pract. 2026 May 8;21:42. doi: 10.1186/s13722-026-00675-1 (PMC13156855; doi:10.1186/s13722-026-00675-1)
Supplement: Supplementary file 1 — Supplementary Material 1 [file 13722_2026_675_MOESM1_ESM.docx]

**Appendix I**

**Network survey of [county name] OUD system community partners**

The [University] is implementing the Continuous and Data-driven Care (CADENCE) program to improve healthcare for pregnant and postpartum individuals with opioid use in [County name], Florida. For this program, the University received funding from the National Institutes of Health under the Helping to End Addiction Long-term (HEAL) Initiative. As a part of the program, we (program team) are conducting a Social Network Analysis to evaluate collaborations among key agencies/ stakeholders involved in providing healthcare and social services such as housing, transportation, food, etc., to pregnant and postpartum individuals with opioid use disorders. As a participating agency, you are requested to diligently fill out the survey required for conducting the Social Network Analysis. Thank you for your agency’s participation in this important initiative. The survey will take approximately **10 – 15 minutes** based on your knowledge of services provided by your agency and not based on your personal experience or work profile.

*If you believe someone else in your agency should fill out the survey, kindly forward it to them.*

**Questions?** Don't hesitate to get in touch with co-PI at [email ID.](mailto:jm@usf.edu) Other approved program team members may contact you on her behalf.

Subjects: You are being asked to participate in this Social Network Analysis survey because you have been serving pregnant and postpartum individuals with a history of opioid use disorder. Your participation is voluntary. Your responses will be anonymous, meaning that we will not identify any participating organization by name, only by service sector.

I agree to participate

Yes – leads to survey

No – leads to end

**…...........................................................................................................................................................**

**Part 1: Agency and Respondent characteristics**

1. Your agency’s name…….
2. Is your agency part of an organization?

Yes If Yes Name …...........................

No

1. What is your role in your agency?

[Add text box]

1. How long have you been working in this agency (in years)?

[Text box to add the number]

1. What services does your agency provide to pregnant and postpartum individuals with opioid use disorder? Check boxes

Prenatal care

Intrapartum care

Postpartum care

Newborn/ Pediatric care

Opioid Use Disorder counselling

Opioid Use Disorder Management

Referral to agencies providing OUD prevention/ management

Provision of safe needles

Legal services

Housing

Transport

Peer-to-peer support groups

Nutrition/ food resources

Ambulance

Child development

Family violence

Others. If yes, enumerate [text box]

1. How many people did you reach/ serve last financial year (Jan 2022-Dec 2022)? (Estimate)

[Text Box]

1. Approximately, what percentage of these people were pregnant or postpartum individuals (Jan 2022-Dec 2022)

[insert slider bar from 0 -100]

1. Approximately, what percent of these pregnant and postpartum individuals had opioid use disorder (Jan 2022-Dec 2022?

[insert slider bar from 0 -100]

**Part 2: The next section will focus on your collaboration with various agencies in the context of addressing opioid use disorder among pregnant and postpartum individuals.**

1. Please select the agency or agencies with which you have an established relationship in the context of addressing opioid use disorder among pregnant and postpartum individuals. **[Enlist all agencies with each agency followed by checkboxes with options for None, Informal, Formal MOU, I don’t know]**

**[Dropdown of all agencies and a few text box options for adding names of agencies]**

*[In Qualtrics, we will add logic here to ensure that the following questions will only have agency names with whom the agency identified an informal or formal relationship in Question 9? For example, if they marked Suncoast and Tampa Bay Thrives as the only agencies, they had a formal/ informal relationship with, from Question 10 to 16, only these two organizations will appear as a list of agencies]*

**Note: *In subsequent questions, we will enquire about your interactions and collaborations with these organizations regarding opioid use disorder in the perinatal period***

1. Which services do you leverage/ seek from these organizations for your clients?

Prenatal care

Intrapartum care

Postpartum care

Newborn/ Pediatric care

Opioid Use Disorder counselling

Opioid Use Disorder Management

Referral to agencies providing OUD prevention/ management

Provision of safe needles

Legal services

Housing

Transport

Peer-to-peer support groups

Nutrition/ food resources

Ambulance

Child development

Family violence

Others. If yes, enumerate [text box]

1. How frequently do you refer pregnant and postpartum individuals with opioid use disorder to the agencies mentioned above [**never, daily, weekly, monthly, quarterly, once in a year**]

**[Dropdown of agencies marked as Informal and Formal MOU in question 9]**

1. How frequently do these agencies refer pregnant/ postpartum individuals with opioid use disorder to your agency **[never, daily, weekly, monthly, quarterly, once in a year]**

**[Dropdown of agencies marked as Informal and Formal MOU in question 9]**

1. How confident are you in each of these agencies for providing services to the clients referred by you **[1 - Not at all confident to 5 - Very confident]** (adapted from <http://assets.thehcn.net/content/sites/arizona/HIPMC_SNA_Report_October_2014_Final.pdf)>

**[Dropdown of agencies marked as Informal and Formal MOU in question 9]**

1. How dependent is your organization on these agencies for achieving your goal of helping pregnant and postpartum individuals with opioid use disorder? **[1 – not at all valuable to 5 – very valuable]** (adapted from <http://assets.thehcn.net/content/sites/arizona/HIPMC_SNA_Report_October_2014_Final.pdf)>
2. How valuable is each organization’s **level of involvement** for your success in achieving your goal of helping pregnant individuals with opioid use disorder? **[1 – not at all valuable to 5 – very valuable]** (adapted from <http://assets.thehcn.net/content/sites/arizona/HIPMC_SNA_Report_October_2014_Final.pdf)>
3. How valuable are this **organization’s services** for your organization’s success in achieving your goal of helping pregnant individuals with opioid use disorder? **[1 – not at all valuable to 5 – very valuable]** (adapted from <http://assets.thehcn.net/content/sites/arizona/HIPMC_SNA_Report_October_2014_Final.pdf)>

**Part 3: The following questions are to understand your organization’s mission and expectations for improving care for people with opioid use disorders.**

1. What is most needed in this community to improve holistic care for people with Opioid Use Disorder [text box]
2. To help us identify are any champions in the community to help people with opioid use disorders, please list the top 3 agencies:
   1. that you go to for information on evidence-based practices for overdose prevention/substance use?

[3 text boxes for the people or organization names]

- 1. who influence local substance use services.

[3 text boxes for the people or organization names]

Note: *A champion can be an individual or organization in your community that you believe has major contributions to helping people with opioid use disorders.*

Thank you for your participation!
